# Protective effects of extracts from Pomegranate peels and seeds on liver fibrosis induced by carbon tetrachloride in rats

**DOI:** 10.1186/s12906-015-0916-9

**Published:** 2015-10-27

**Authors:** Xiang-lan Wei, Ru-tang Fang, Yong-hua Yang, Xue-yuan Bi, Guo-xia Ren, A-li Luo, Ming Zhao, Wei-jin Zang

**Affiliations:** Department of Pharmacology, Xi’an Jiaotong University Health Science Center, P.O. Box 77#, No.76 Yanta West Road, Xi’an, 710061 People’s Republic of China; Department of Pharmacy, Xi’an Chest Hospital, Xi’an, 710061 People’s Republic of China; Department of Gastroenterology, The 451 Hospital of the Chinese People’s Liberation Army, Xi’an, 710054 People’s Republic of China; Department of pediatrics, The First Affiliated Hospital of Xi’an Jiaotong University, Xi’an, 710061 People’s Republic of China

**Keywords:** The extract of pomegranate peels, The extract of pomegranate seeds, Liver fibrosis, Oxidative stress

## Abstract

**Background:**

Liver fibrosis is a feature in the majority of chronic liver diseases and oxidative stress is considered to be its main pathogenic mechanism. Antioxidants including vitamin E, are effective in preventing liver fibrogenesis. Several plant-drived antioxidants, such as silymarin, baicalin, beicalein, quercetin, apigenin, were shown to interfere with liver fibrogenesis. The antioxidans above are polyphenols, flavonoids or structurally related compounds which are the main chemical components of Pomegranate peels and seeds, and the antioxidant activity of Pomegranate peels and seeds have been verified. Here we investigated whether the extracts of pomegranate peels (EPP) and seeds (EPS) have preventive efficacy on liver fibrosis induced by carbon tetrachloride (CCl_4_) in rats and explored its possible mechanisms.

**Methods:**

The animal model was established by injection with 50 % CCl_4_ subcutaneously in male wistar rats twice a week for four weeks. Meanwhile, EPP and EPS were administered orally every day for 4 weeks, respectively. The protective effects of EPP and EPS on biochemical metabolic parameters, liver function, oxidative markers, activities of antioxidant enzymes and liver fibrosis were determined in CCl_4_-induced liver toxicity in rats.

**Results:**

Compared with the sham group, the liver function was worse in CCl_4_ group, manifested as increased levels of serum alanine aminotransferase, aspartate aminotransferase and total bilirubin. EPP and EPS treatment significantly ameliorated these effects of CCl_4_. EPP and EPS attenuated CCl_4_–induced increase in the levels of TGF-β1, hydroxyproline, hyaluronic acid laminin and procollagen type III. They also restored the decreased superoxide dismutase (SOD), glutathione peroxidase (GSH-Px) activities and inhibited the formation of lipid peroxidized products in rats treated with CCl_4_.

**Conclusion:**

The EPP and EPS have protective effects against liver fibrosis induced by CCl_4_, and its mechanisms might be associated with their antioxidant activity, the ability of decreasing the level of TGF-β1 and inhibition of collagen synthesis.

## Background

Liver fibrosis which is a common feature of the majority of chronic liver diseases is considered as an important medical problem due to its high morbidity and mortality [1, 2]. Hepatitis viral infections are the most common cause, and drug abuse, autoimmune disorders, biliary obstruction and metabolic disorders due to mineral overload are also contributed to the disease [3, 4], which is characterized as excessive accumulation of collagen and extracellular matrix proteins within the perisinusoidal space [5, 6]. Advanced liver fibrosis could leads to cirrhosis and liver failure eventually. Now there is few effective medical treatments approaches for liver fibrosis [6].

The pathogenesis of liver fibrosis remains elusive. Oxidative stress is recognized as a fundamental factor in the pathological changes observed in various liver diseases [7, 8]. Free radicals, formed as a result of oxidation, attack the unsaturated fatty acids of biomembranes, which results in peroxidation and destruction of protein and DNA, leading to various liver injuries [9]. Several lines of evidence have recently suggested that oxidative stress plays a pivotal role in the pathogenesis of liver fibrosis [10]. Antioxidants are effective for preventing liver fibrogenesis [11–13].

Pomegranate belongs to the Punicagranatum L., and is widely cultivated in India, Southeast Asia, Malaya, the East Indies, tropical Africa and China [14, 15]. As in the earlier study, pomegranate had been used as an anthelmintic and antidiarrheal agent. Recently, the antioxidant activity of pomegranate is more and more being paid attention [16]. Pomegranate contains large amounts of polyphenols and flavonoid, so that the antioxidant capacity are obvious among in pomegranate fruit, juice and peel [17–20]. There is growing evidence that EPP have effective effect against liver fibrosis in rats. Experimental observations indicated that punicagranatum peel methanolic extract reversed thioacetamide-induced liver fibrosis, and significantly decreased the activity of liver enzymes, bilirubin and serum hepatocyte growth factor levels. These effects could be attributed to its antioxidant properties, antifibrotic and antiapoptotic activity [21]. Tokluet al. have also found that EPP prevents liver fibrosis in biliary-obstructed rats, and forecasted EPP’s antioxidant and antifibrotic properties, may be of potential therapeutic value in protecting the liver from fibrosis and oxidative injury due to biliary obstruction [22]. However, the antifibrotic effect of EPS on chronic liver diseases has not been published in the literature. In this study, we aimed to investigate the effect of EPP and EPS on CCl_4_-induced liver fibrosis in rats and to propose its possible mechanism.

## Methods

### Chemicals

Carbon tetrachloride (CCl_4_) was purchased from Sigma (St. Louis,MO, USA). Commercial kits for aspartate aminotransferase (AST), alanine aminotransferase (ALT), total bilirubin (T-Bil), malondialdehyde (MDA), glutathione peroxidase (GSH-Px), and superoxide dismutase (SOD), as well as hydroxyprolinewere purchased from Jiancheng Biotechnology Company (Nanjing, China). The hyaluronic acid (HA), Laminin (LN), and procollagen type III (PC III) radioimmunoassay kits were purchased from Beijing North Instirute of Biotechnology (Beijing, China). Transforming growth factor-β1 (TGF-β1) was purchased from Shanghai Huole Institute of Biotechnology (Shanghai, China). Allother chemicals and reagents used in this study were of analyticalgrade.

### The extracts of pomegranate peels and seeds preparation

Fresh pomegranate fruits were collected in September to October, 2011 from a natural habitat in Shannxi, China. Then the pomegranate peels and seeds were artificially isolated and cleaned, air-dried. The dried pomegranate peels and seeds were grinded to coarse powder and passed through the 60 mesh screen, respectively. The above materials and methods were authenticated by Professor Qiang Wang who works at Department of Chinese Materia Medica Analysis, China Pharmaceutical University, Nanjing, where a voucher specimen has been deposited (2011–09).

The EPP and EPS were prepared following the method described previously with some modification [23]. The 3 kilogramme pomegranate peel powder and the 2 kilogramme pomegranate seed powder were extracted with 50 % ethanol in triplicate at 85 °C, respectively. The extracts were then filtered and evaporate under vacuum until dried. The dried EPP and EPS were stored in a refrigerator.

### Animals

The forty male wistar rats aged 6–8 weeks old and weighed 180–200 g purchased from the Experimental Animal Center Jiaotong University. Rats were housed in plastic cages (five rats per cage) at 24 °C and 60 % humidity with a 12 h-12 h light–dark cycle and ad libitum access to water and standard rat chow. The study protocol was approved by the Ethics committee of the Xi’an Jiaotong University Health Science Center. Animal handling and care was carried out throughout the experiment following international laboratory animal use and care guidelines (NIH publication no. 85–23, revised1996).

### Experimental model and drug treatment

In this experiment, the rats were randomly divided into four groups, each of whom has ten rats. (1) Sham group (sham), in which rats were subcutaneously injected with vegetal oil without CCl_4_ (1 mg/kg body weight, twice a week for four weeks; (2) CCl_4_ group (CCl_4_), in which rats were subcutaneously injected with 50 % CCl_4_ mixed with vegetal oil (1 mL/kg body weight) [24, 25]; (3) CCl_4_ + EPP group (CCl_4_ + EPP), in which rats were administered with EPP (150 mg/kg body weight) intragastrically every day for 4 weeks at the same time subjected to CCl_4_ and (4) CCl_4_ + EPS group (CCl_4_ + EPS), in which rats were administered EPS (100 mg/kg body weight) intragastrically every day for 4 weeks at the same time subjected to CCl_4_. At 24 h after the final injection of CCl_4_, all rats were anaesthetized with diethyl ether, and the blood samples were collected from the orbital veins. The serum samples were obtained by centrifugation of blood at 3000 rpm for 15 min and were kept frozen at −80 °C until assayed as described below. After the rats were killed, the livers and spleen were immediately removed, rinsed in ice-cold saline and weighed. A portion of each liver was fixed in 10 % formalin for histological analyses, and the remaining tissues were stored at −80 °C until required.

### Liver index and spleen index

Relative weights of liver and spleen were expressed as the percentage of the body weight respectively.

### Biochemical metabolic parameters

#### Assessment of liver function

The serum activities of AST and ALT were evaluated by spectrophotometry using commercially available kits according to the manufacturer's instructions; The levels of HA, LN and PC III were determined using radioimmunoassay kits according to the manufacturer’s instructions; TGF-β1 level in serum was deternined by using rat enzyme-linked immunosorbent assay (ELISA) kit.

#### Assessment of oxidative stress and enzymatic antioxidant status

The tissue homogenates (10 %, w/v) of livers were used in determination of MDA, SOD and GSH-Px levels according to the instructions.

#### Assessment of collagen

Liver collagen concentration was determined by measuring Hydroxyproline level according to the method described previously [1].

### Histological observation

Rat livers were carefully isolated and fixed with 10 % formalin solution for 24 h. Fixed tissues were dehydrated and embedded in paraffin and then cut into 5 μm sections with a Leica RM2135 (Leica, Bensheim, Germany) for staining. Sections were stained with Hematoxylin-eosin staining (HE) to observe liver injury, stained with Masson staining to detect collagen deposition and stained with Sirius red stain to measure the collagen content and type. The pathological changes were assessed and photographed with an Nikon, Japan microsco Liver fibrosis equipped with digital camera (Nikon, Japan) or polarization microscope (Nikon, Japan). was graded according to the following scoring system as following [26]: 0, no fibrosis, normal liver and absence of fibrosis; I, fibrosis present (collagen fiber present that extends from portal triad or central vein to peripheral region); II, mild fibrosis (mild collagen fiber present with extension without compartment formation); III, moderate fibrosis (moderate collagen fiber present with some pseudo lobe formation) and IV, severe fibrosis (severe collagen fiber present with thickening of the partial compartments and frequent pseudo lobe formation).

### Statistical analysis

Quantitative data was expressed as the mean ± S.D. The significance of the difference versus the CCl_4_ group was determined by a Student’s *t*-test. A Mann-Whitney rank sum test was used for the analysis of degree of histopathological liver fibrosis. *P* < 0.05 was considered to be statistically significant.

## Results

### The effects of extracts on body weight, liver and spleen index

The effects of EPP and EPS on the rats’ body weight were evaluated. Rats were subcutaneously injected with CCl_4_, twice a week for four weeks. The body weight gain were statistically reduced compared with the sham group (92.74 ± 18.61 vs. 141.09 ± 12.72 g, *P* < 0.05, *n* = 10). Pretreatment with either EPP or EPS increase the final body weights of rats to 308.22 ± 19.44 g and 309.10 ± 20.72 g respectively. Their body weight gain were higher than CCl_4_ group (128.30 ± 24.50 and 125.10 ± 22.09 g, vs. CCl_4_ group, *P* < 0.05, *n* = 10, Table 1).

**Table 1:** Initial body weight, final body weight, body weight gain, liver index and spleen index in differente experimental groups

Groups	Body weight (g)		Body weight gain (g)	Liver index (%)	Spleen index (%)
	Initial	Final			
Sham	180.41 ± 14.31	321.50 ± 18.49	141.09 ± 12.72	2.75 ± 0.42	0.25 ± 0.035
CCl_4_	179.66 ± 10.10	272.40 ± 21.03	92.74 ± 18.61*	5.97 ± 0.49^*^	0.50 ± 0.055^*^
CCl_4_ + EPP	180.82 ± 9.28	308.22 ± 19.44	128.30 ± 24.50^#^	3.58 ± 0.63^#^	0.36 ± 0.055^#^
CCl_4_ + EPS	184.00 ± 10.98	309.10 ± 20.72	125.10 ± 22.09^#^	3.55 ± 0.55^#^	0.35 ± 0.039^#^

EPP (150 mg/kg) and EPS (100 mg/kg) were administered orally for 4 weeks. The values are expressed as the mean ± S.D. (*n* = 10).*P < 0.05, compared with the sham group, ^*#*^ *P* < 0.05, compared with the CCl_4_ group

Subsequently, the liver and spleen index were calculated as the percentage of the body weight. The results showed that the liver and spleen index in CCl_4_ group were significantly higher than the sham group (5.97 ± 0.49 and 0.50 ± 0.055 % vs. 2.75 ± 0.42 and 0.25 ± 0.035 %, *P* < 0.05, *n* = 10). While fed with the EPP and EPS, these indexes were significantly lower than the CCl_4_ group (EPP: 3.58 ± 0.63 and 0.36 ± 0.055 %; EPS: 3.55 ± 0.55 and 0.35 ± 0.039 %, vs. CCl_4_ group, *P* < 0.05, *n* = 10, Table 1).

### The effects of extracts on liver function

**Fig. 1:**
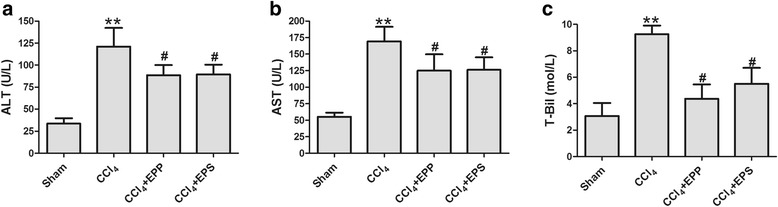
Effects of EPP and EPS on serum of ALT (**a**), AST (**b**) and T-Bil (**c**) after CCl_4_ injury. **P < 0.01, vs. sham group;^*#*^ *P* < 0.05, vs. CCl_4_ group; *n* = 10 in each group; values are mean ± S.D

The significantly higher levels of ALT, AST and T-Bil in the serum of the rats in the CCl_4_ group were exhibited than that of the sham group (ALT: 121.17 ± 21.24 vs. 33.70 ± 6.04 U/L; AST: 169.20 ± 22.15 vs. 55.31 ± 6.22 U/L; T-Bil: 9.25 ± 0.66 vs. 3.07 ± 0.98 mol/L, respectively; *P* < 0.01, *n* = 10). EPP and EPS treatment efficiently reduced the ALT level to 88.50 ± 11.62 and 89.62 ± 11.01 U/L (*P* < 0.05, *n* = 10, Fig. 1a), decreased the AST to 124.90 ± 24.90 and 126.33 ± 18.84 U/L (*P* < 0.05, *n* = 10, Fig. 1b), and also significantly suppressed CCl_4_–induced increase in T-Bil level (4.73 ± 1.09 and 5.51 ± 1.20 mol/L, *P* < 0.05, *n* = 10, Fig. 1c). The result indicated that the EPP and EPS had protective effects against liver injuries in rats injected with CCl_4_.

### The effects of extracts on the microstructure of liver

**Fig. 2:**
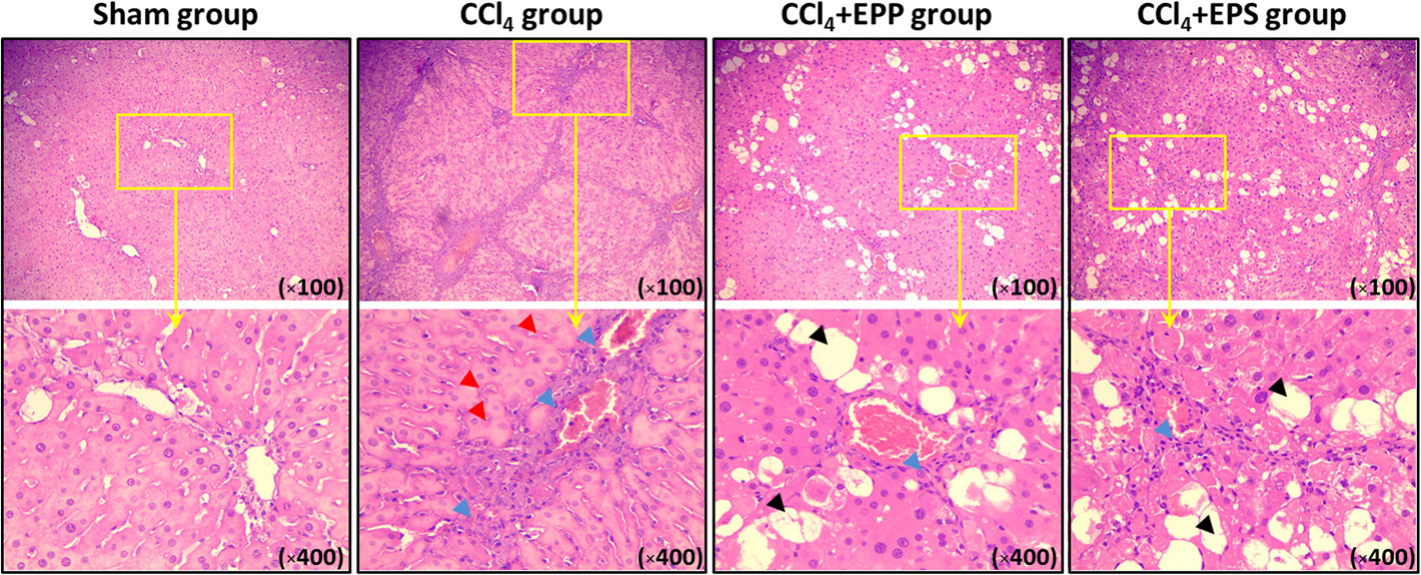
Effects of EPP and EPS on the microstructure of CCl_4_ injury liver. HE staining analysis of hepatic histological abnormalities showing hepatocellular degeneration (blue arrowheads), infiltration inflammatory cells (red arrowheads) and vacuolated cells (black arrowheads)

The effects of EPP and EPS on the microstructure changes of liver were shown in Fig. 2. The results of HE staining indicated that CCl_4_ administration presented extensive changes in liver morphology, including marked enlarged areas of hepatocellular degeneration and infiltration inflammatory cells. No histological abnormalities were observed in sham group. EPP and EPS treatment significantly alleviated the degree of liver injury.

### The EPP and EPS attenuates CCl_4_-induced liver fibrosis

**Fig. 3:**
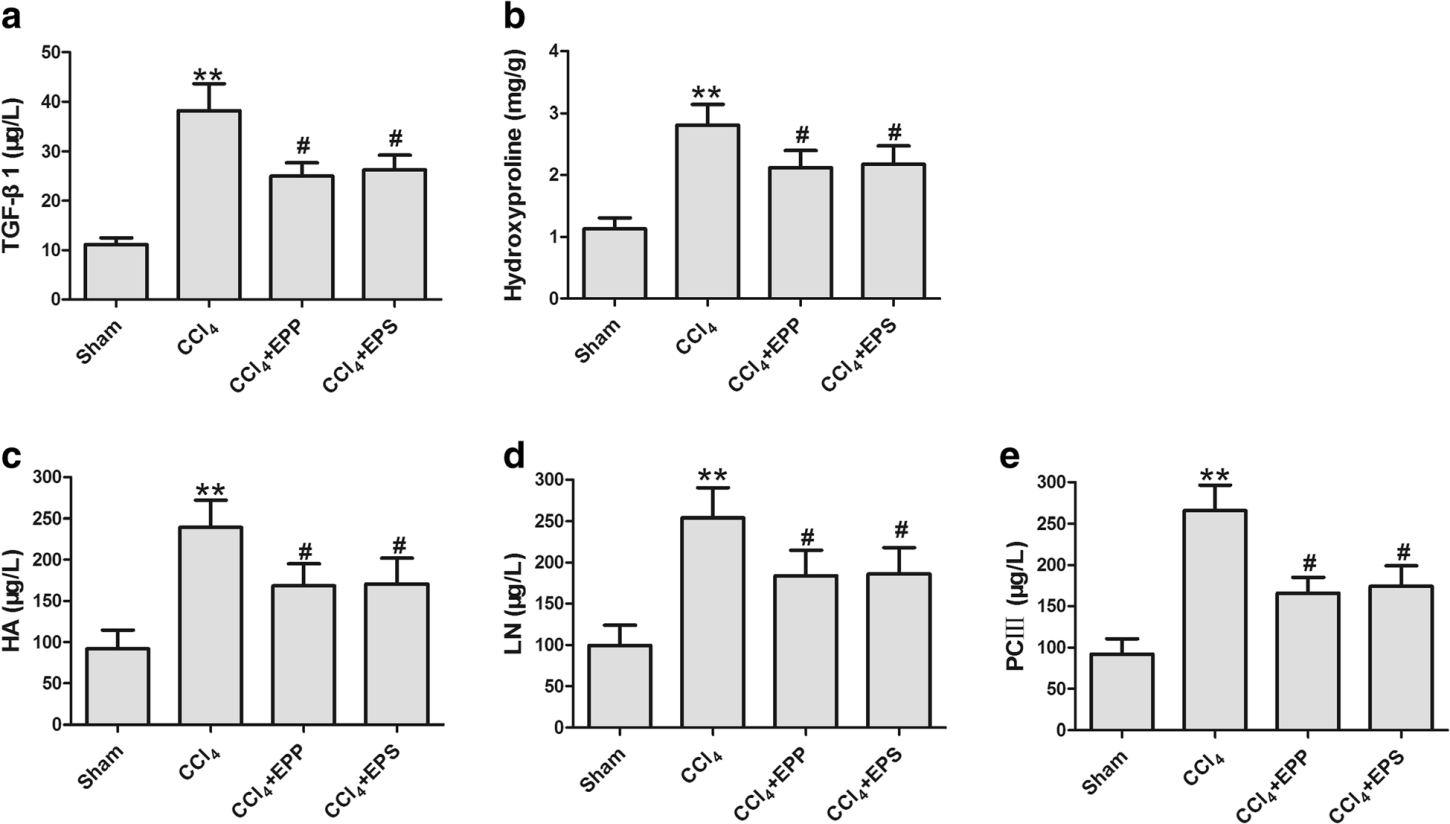
Effects of EPP and EPS onserum of TGF-β1 (**a**), HA (**c**), LN (**d**), PC III (**e**) and liver of hydroxyproline (**b**) after CCl_4_ injury.**P < 0.01, vs. sham group; ^***#***^ *P* < 0.05, vs. CCl_4_ group; *n* = 10 in each group; values are mean ± S.D

Further, the effects of EPP and EPS on the markers of liver fibrosis were examined. As shown in Fig. 3a, the level of TGF-β1 in CCl_4_ group was elevated significantly compared to that of the sham group (38.27 ± 5.37 vs. 11.14 ± 1.39 μg/L, *P* < 0.01, *n* = 10). EPP and EPS significantly decreased the TGF-β1 level in serum compared to that of the CCl_4_ group (25.00 ± 2.67 and 26.29 ± 2.95 μg/L,*P* < 0.05, *n* = 10).

Hydroxyproline content in liver and serum levels of HA, LN and PC III as the indexes of liver fibrosis were tested. It is shown that CCl_4_ significantly increased hydroxyproline, HA, LN and PC III compare with that of the sham group (hydroxyproline: 2.81 ± 0.33 vs. 1.13 ± 0.18 mg/g; HA: 239.38 ± 32.80 vs. 92.08 ± 22.77 μg/L; LN: 253.85 ± 36.47 vs. 99.54 ± 24.63 μg/L and PC III: 256.26 ± 31.40 vs. 92.17 ± 18.48 μg/L, *P* < 0.01, *n* = 10). EPP and EPS had potential for the reduction of hydroxyproline, HA, LN and PC III level that increased by CCl_4_. The final values were hydroxyproline: 2.12 ± 0.28 and 2.17 ± 0.30 mg/g; HA: 168.64 ± 26.61 and 170.98 ± 31.11 μg/L; LN: 183.77 ± 31.12 and 186.17 ± 31.95 μg/L; PC III: 165.77 ± 19.30 and 174.30 ± 24.93 μg/L (*P* < 0.05, *n* = 10, fig B-E).

### The effects of extracts on liver fibrosis histological examination

**Fig. 4:**
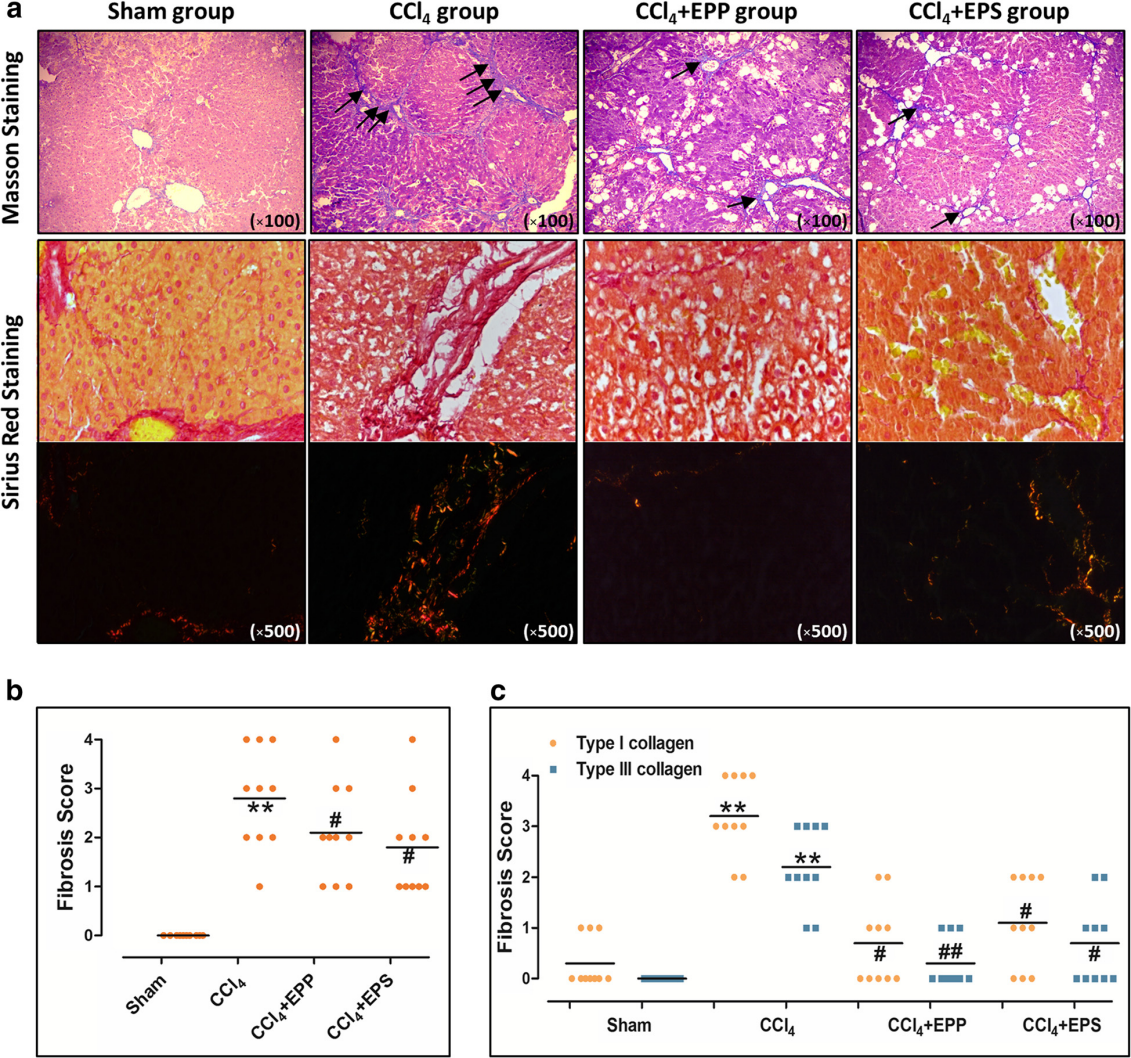
Effect of EPP and EPS on histological morphology of fibrosis rat liver with Masson and Sirius red staining. **a**: Masson and Sirius red staining analysis of hepatic fibrosis (arrows); **b**: the graded of fibrosis with Masson staining; **c**: the type and graded of fibrosis with Sirius red staining.**P < 0.01, vs. sham group; ^***##***^ *P* < 0.01,^***#***^ *P* < 0.05, vs. CCl_4_ group; *n* = 10 in each group; values are mean ± S.D

Liver fibrosis was evaluated histologically using Masson staining and Sirius red staining in the different groups. The Masson staining showed that the normal hepatic lobules in CCl_4_ group were disappeared, collagen was deposited and pseudo lobule was formed. Treatment with EPP and EPS obviously alleviated the collagen deposition. Fig. 4b showed that treatment with EPP and EPS significantly lower the graded of fibrosis as compared to CCl_4_ group (*P* < 0.01, *P* < 0.05, *n* = 10).

Sirius red stains collagen red on a pale yellow background in brightfield microscope, whereas under polarization microscope the typeI collagen appears bright orange-red and the type III collagen appears bright yellow-green. Sirius red staining showed that in CCl_4_ group, type I and III collagen were more than that of the sham group, and treatment with EPP and EPS could effectively against the increase in type I and III collagen induced by CCl_4_ (*P* < 0.01, *P* < 0.05, *n* = 10, Fig. 4c).

### The effects of extracts on hepatic MDA, SOD and GSH-Px activities

**Fig. 5:**
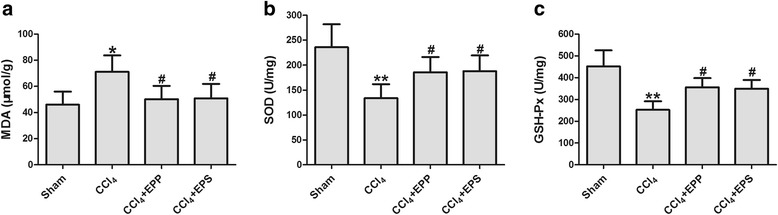
Effects of EPP and EPS on MDA level (**a**), SOD (**b**) and GSH-Px (**c**) activities in liver homogenates of liver fibrosis rats. **P < 0.01,*P < 0.05 vs. sham group;^***#***^ *P* < 0.05, vs. CCl_4_ group; *n* = 10 in each group; values are mean ± S.D

As expected, the treatment with EPP and EPS also showed effective anti-oxygenation in CCl_4_ injury rats. MDA levels, and the end product of lipid peroxidation, were significantly higher in the CCl_4_ group than in the sham-operated group (*P* < 0.05). In the EPP and EPS treated groups, hepatic MDA levels were markedly decreased compared with that of the CCl_4_ group (EPP: 50.21 ± 10.23 vs. 71.10 ± 12.63 μmol/g; EPS: 50.84 ± 11.05 μmol/g, *P* < 0.05, *n* = 10, Fig. 5a).

Meanwhile, thehepatic SOD and GSH-Pxactivities were lower in the CCl_4_ group than in the sham-operated group (*P* < 0.01). EPP and EPS treatment both efficiently increased the SOD activities from 133.47 ± 28.29 U/mg in the CCl_4_ group to 185.72 ± 30.05 U/mg in the EPP group and 187.94 ± 21.38 U/mg in the EPS group (*P* < 0.05, *n* = 10, Fig. 5b). They also efficiently increased the GSH-Pxactivities from 252.07 ± 39.39 U/mg in the CCl_4_ group to 355.73 ± 41.88 U/mg in the EPP group and 349.47 ± 40.10 U/mg in the EPS group (*P* < 0.05, *n* = 10, Fig. 5c).

## Discussion

The aim of this study is to evaluate the protective effects of the extracts of the pomegranate peels and seeds against liver fibrosis induced by CCl_4_ in rats. The study showed that EPP and EPS treatment appeared to be beneficial in this animal model of CCl_4_ injury in terms of reduced liver index and spleen index, improved liver function, and inhibited the development of hepatic fibrosis rats. These protective effects may depend on decreased MDA levels and increase SOD and GSH-Px activities, which implied anti-oxygenation activity may be the possible mechanism of EPP and EPS in abrogating liver fibrosis.

CCl_4_ treatment is frequently used in rats to produce an experimental model to study liver fibrosis [27, 28]. In the present study, liver fibrosis was successfully induced by subcutaneous injection of 50 % CCl_4_ for a period of 4 weeks. The histological results showed that the normal structure of lobules was destroyed and pseudolobules formed. Moreover, the increased hydroxyproline content in liver and serum HA, LN and PC III also confirmed the liver fibrosis in rats.

The increased levels of aminotransferases are the results of leakage from damaged hepatic cells and are used as markers of liver injury [29]. It was shown in this study that abnormal aminotransferase activity was noted in rats injected with CCl_4_ and were decreased by administration of the EPP and EPS. These results provided evidence that the EPP and EPS are able to improve hepatic steatosis in rats without obvious hepatotoxicity. The hepato protective effects of EPP and EPS were further evidenced by marked improvement in the histopathological examination.

Proteins HA, LN and PC III are important biomarkers of hepatic fibrogenesis [30, 31]. Hydroxyproline in liver is an important index whose level reflects the amount of collagen and therefore it can be used to determine the extent of fibrosis [32]. Thus, HA, LN, PC III and hydroxyproline are important indexes to appraisal liver fibrosis. The result in this study showed that EPP and EPS could significantly decrease HA, LN and PC III in serum and hydroxyproline content in liver, which suggested that EPP and EPS can inhibit collagen deposition in liver.

Being recognized as a major profibrogenic cytokine in the progression of liver fibrosis, TGF-β1 is responsible for hepatic stellate cells (HSCs) activation and migration which is supposed to be the central event of liver fibrosis [33, 34]. It has been reported that anti-TGF-β1 antibody ameliorated concanavalin A-induced liver fibrosis, while over-expression of TGF-β1 in transgenic mice resulted in the acceleration of liver fibrosis progression [35, 36]. The overexpression of TGF-β1 has been correlated with the degree of fibrosis in both animal models and human diseases. The results in this study showed that EPP and EPS decreased the serum levels of TGF-β1, which suggested that these extracts may be associated with the regulation of cytokine release.

It has been proved that oxidative stress plays an important role in the generation of CCl_4_-induced liver fibrosis [37–39]. CCl_4_ can be metabolized by cytochrome P450 in liver cells after being injected into rats, and eventually yield the trichloromethyl-free radical. These compounds trigger a cascade of reactions and lipid peroxidation, which lead to cell injury and chronic liver injury with the result of liver fibrosis [40]. MDA is the main product of lipid peroxidation and its concentration is generally presented as the total level of lipid peroxidation products [41]. SOD and GSH-Px are key enzymes in antioxidant defense system, and they can catalyze the transformation of hydrogen peroxide into water [42–44]. The results in this study demonstrated that CCl_4_ led to increases of MDA levels and decreases of SOD and GSH-Px levels in the liver. Administration of EPP and EPS increased SOD and GSH-Px activity, while depressed MDA production in hepatic tissues in rats treated with CCl_4_. These results indicated that the protective effects of EPP and EPS on liver fibrosis were partly due to their antioxidant activities.

Additionally, this study showed that EPP and EPS affected the protective action on liver injury and antioxidant state. These resulte are probably attributed to its phenolic compounds and linolenic acid contained in this fruit. EPP mainly contain total phenolics, including mainly hydrolyzable tannins (ellagitannins), such as oligomers and punicalagin/punicalin. Nevertheless, EPS mainly contain fatty acids (eg. punicic acid, linolenic acid isomers, linoleic, palmitic and stearic, et al.), no phenolics are present [22]. The antioxidant activity of phenolics and linolenic acid are mainly due to their redox properties, which allow them to act as reducing agents, hydrogen donors, singlet oxygen quenchers, and metal chelators [22, 45].

## Conclusions

In conclusion, pomegranate is a high nutritious and desirable fruit with rich bioactive profile. Because of its beneficial physiological activities, the consumption of pomegranate has grown tremendously. The present study showed that pomegranate peels and seeds have preventive effects against liver fibrosis. The main mechanisms of the effects might be related to its antioxidant activity, decreased the level of TGF-β1 and inhibition of collagen synthesis. However, further studies are required.
